# Knee positioning systems for X-ray environment: a literature review

**DOI:** 10.1007/s13246-023-01221-y

**Published:** 2023-01-24

**Authors:** Catarina Lopes, Adelio Vilaca, Cláudia Rocha, Joaquim Mendes

**Affiliations:** 1 Faculty of Engineering, University of Porto, Porto, Rua Dr. Roberto Frias, 4200-465 Porto, Portugal; 2 Abel Salazar Biomedical Sciences Institute, University of Porto, Porto, Rua Jorge de Viterbo Ferreira, 4050-313 Porto, Portugal; 3 Department of Orthopaedics, Hospital de Santo António (HSA), Centro Hospitalar Universitário do Porto (CHUPorto), Porto, Largo do Professor Abel Salazar, 4099-001 Porto, Portugal; 4 Centre for Robotics in Industry and Intelligent Systems, Institute for Systems and Computer Engineering, Technology and Science (INESC TEC), Porto, Rua Dr. Roberto Frias, 4200-465 Porto, Portugal; 5 Institute of Science and Innovation in Mechanical and Industrial Engineering (INEGI), Associated Laboratory for Energy, Transports and Aeronautics (LAETA), Rua Dr. Roberto Frias 400, 4200-465 Porto, Portugal

**Keywords:** Knee joint, Lachman test, Positioning system, Stress radiography, Valgus stress, Varus stress

## Abstract

The knee is one of the most stressed joints of the human body, being susceptible to ligament injuries and degenerative diseases. Due to the rising incidence of knee pathologies, the number of knee X-rays acquired is also increasing. Such X-rays are obtained for the diagnosis of knee injuries, the evaluation of the knee before and after surgery, and the monitoring of the knee joint’s stability. These types of diagnosis and monitoring of the knee usually involve radiography under physical stress. This widely used medical tool provides a more objective analysis of the measurement of the knee laxity than a physical examination does, involving knee stress tests, such as valgus, varus, and Lachman. Despite being an improvement to physical examination regarding the physician’s bias, stress radiography is still performed manually in a lot of healthcare facilities. To avoid exposing the physician to radiation and to decrease the number of X-ray images rejected due to inadequate positioning of the patient or the presence of artefacts, positioning systems for stress radiography of the knee have been developed. This review analyses knee positioning systems for X-ray environment, concluding that they have improved the objectivity and reproducibility during stress radiographs, but have failed to either be radiolucent or versatile with a simple ergonomic set-up.

## Introduction

Every year, worldwide, there are several injuries that affect the human knee joint, and its ligaments, originating joint instability. The assessment of this instability is not an easy process for physicians to quantify. Therefore, there is a growing need to have systems to automate an objective diagnosis.

The knee is the largest and, arguably, the most stressed joint of the human body [17, 25, 33]. It is a compound synovial joint that essentially serves as a hinge, meaning that it primarily allows movement along one axis in terms of flexion and extension of the knee in the sagittal plane, with limited motion along other planes [17, 19, 25, 33]. This type of motion makes the knee joint an essential component of efficient bipedal movements, such as walking, running, and jumping [17]. Since bipedal movements bring upon the knee considerable amounts of biomechanical stress, stability of this joint is provided by the arrangement of its ligaments and the extension of muscles that cross the joint [17, 25]. Having minimal side-to-side motion, the knee is prone to injury, especially ligament injuries. The most common knee ligament injuries are in the anterior cruciate ligament (ACL) and the medial collateral ligament (MCL), which are two important stabilising components of the knee joint. The ACL is usually injured during sports and fitness activities that can put stress on the knee, or by trauma victims who tear or sprain the ACL [3, 5]. The majority of MCL tears are isolated and occur in individuals that participate in sports activities involving knee flexion and/or contact [40, 46]. Apart from ligament injuries, the knee joint is most commonly affected by knee osteoarthritis (KOA), which is a degenerative knee joint disease. KOA is typically the result of wear and tear and progressive loss of articular knee cartilage [11, 20]. The most common treatments for knee injuries depend on the severity of the injury. These treatments can either be non-operative or operative. The non-operative treatments involve medication and rehabilitation, whereas the operative treatments involve surgery that could go as far as total knee replacement [20].

**Fig. 1:**
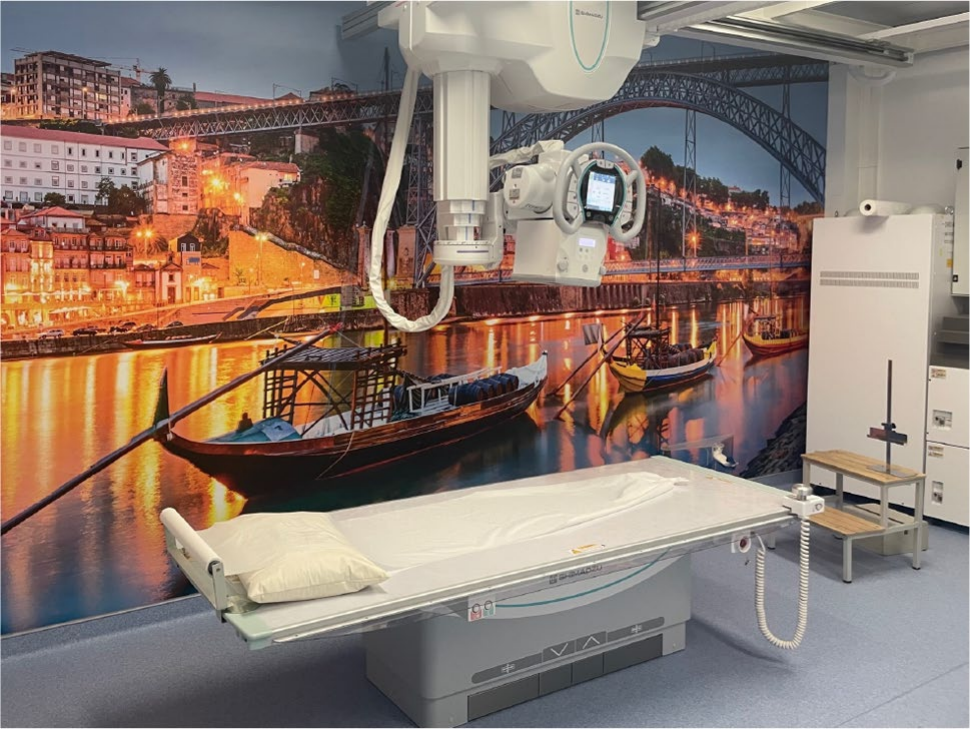
X-ray equipment

**Fig. 2:**
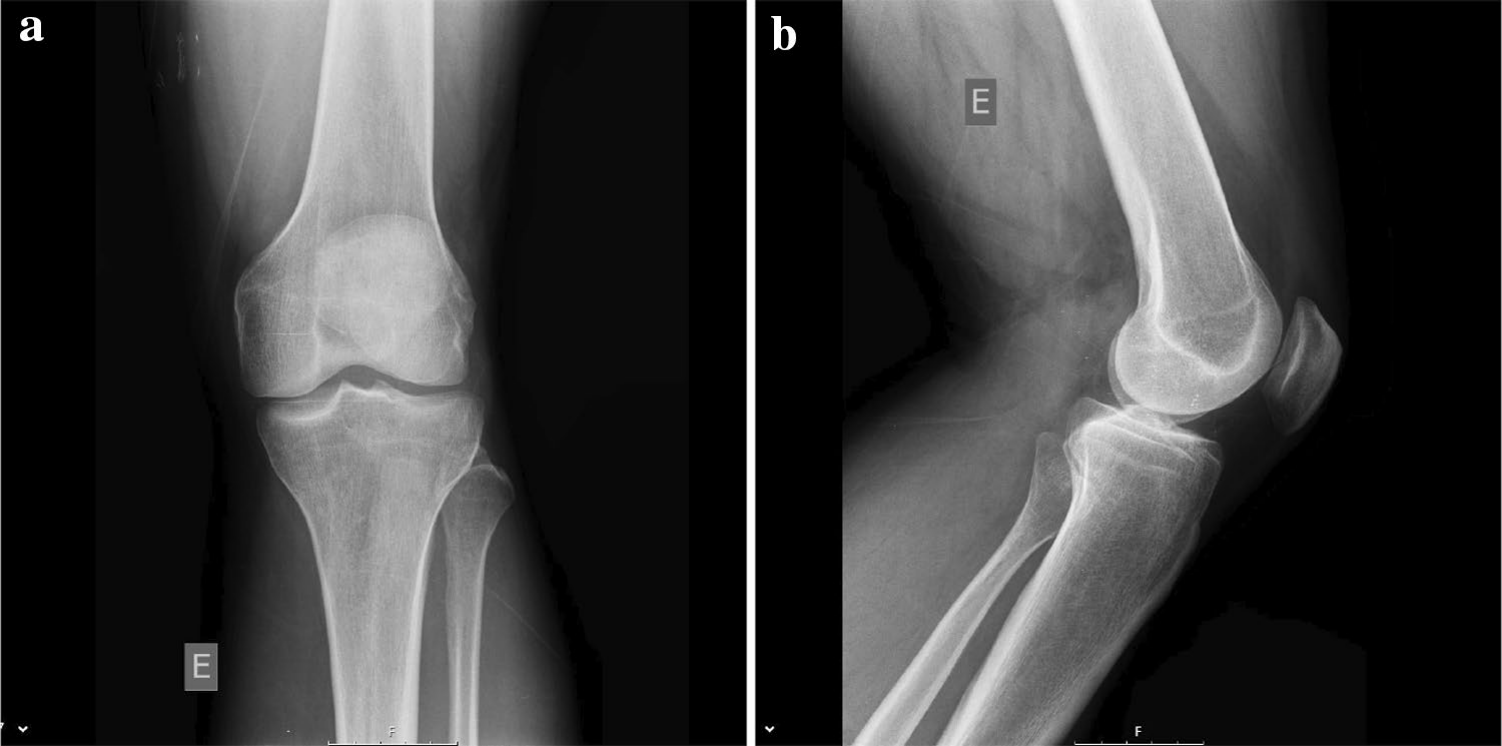
Left knee X-rays, where “E” is an indicator for “Left”: **a** normal anteroposterior view, and **b** lateral view

Radiography is a widely used medical tool for the aforementioned knee pathologies. It is a broad term that addresses different types of medical examinations that use an X-ray approach to visualise the internal structure of a patient’s body [9]. However, radiography is commonly used when referring to a plain X-ray—the first-line investigation for suspected orthopaedic pathology, even before other widely used imaging techniques, such as computed tomography (CT), and magnetic resonance imaging (MRI) [38]. X-ray is the gold standard and an accurate mean of evaluating not only the bone, but also its relationship to orthopaedic structure. Moreover, it is used for the diagnosis and treatment of patients by the passage of an X-ray beam through their body [9, 38]. A portion of the X-rays are absorbed or scattered by the body, whereas the remaining X-rays are transmitted to a detector creating a high-resolution two-dimensional image [9]. This is because different tissues will absorb different amounts of radiation leading to the well-known grey-scaled X-ray images [47]. In spite of the images generated by CT or MRI scans being in 3D and, therefore, more detailed, the X-ray is usually the first technique chosen, since it is a faster and cheaper way of diagnosis and monitoring that already shows indirect signs of ligament ruptures or KOA [14]. Figure 1 exemplifies an X-ray equipment, whereas Fig. 2 shows images of knee X-rays.

In 2020, 87 million of patients worldwide, over 20 years old, suffered from KOA [11]. In the United States alone there are between 100,000 and 200,000 ACL ruptures per year [3, 5], and around 74,000 MCL injuries per year [32, 46]. These numbers are expected to increase since there has been a slow rise in the incidence of KOA due to the ageing of the population [34, 39, 45], and of knee ligament injuries in young people related to high-intensity sports with lack of injury prevention [1, 18, 49]. With the rising incidence of these pathologies, the number of X-rays for diagnosis of knee injuries, and for evaluation/monitoring of knee stability will increase.

**Fig. 3:**
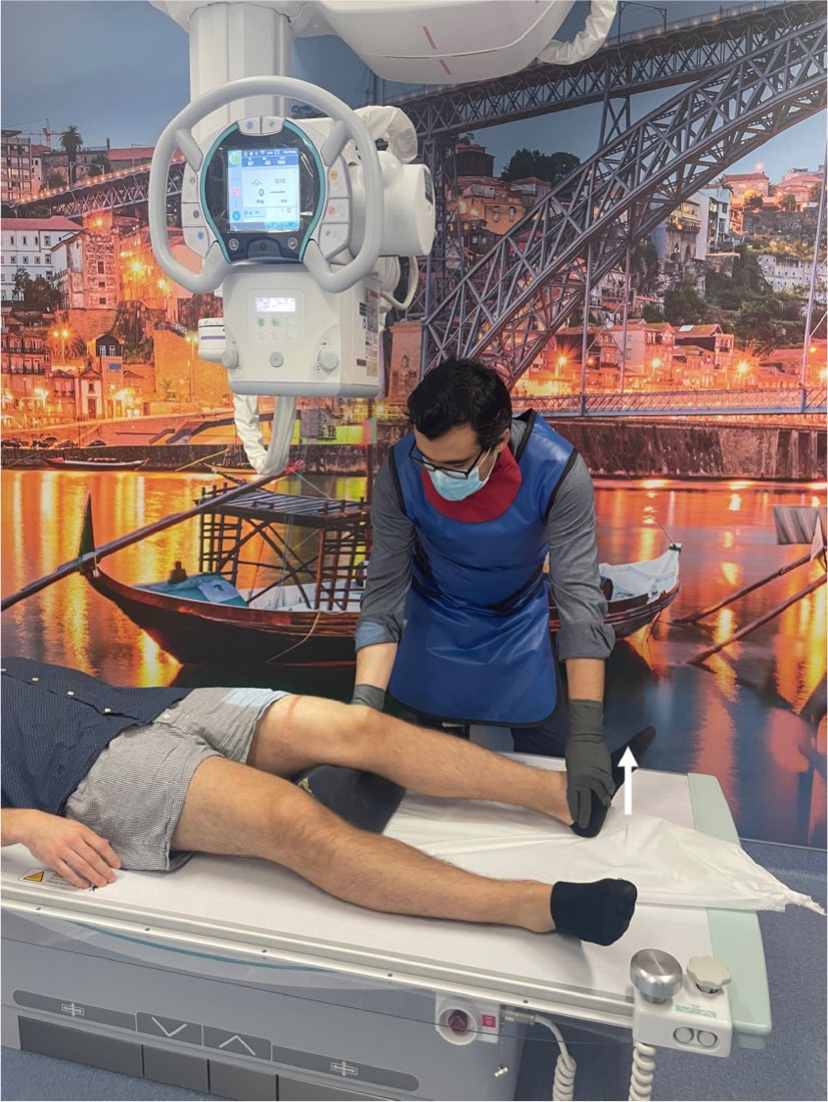
Valgus stress test performed manually by a physician. The arrow indicates the movement of the hand on the tibia whilst the other hand fixes the femur

**Fig. 4:**
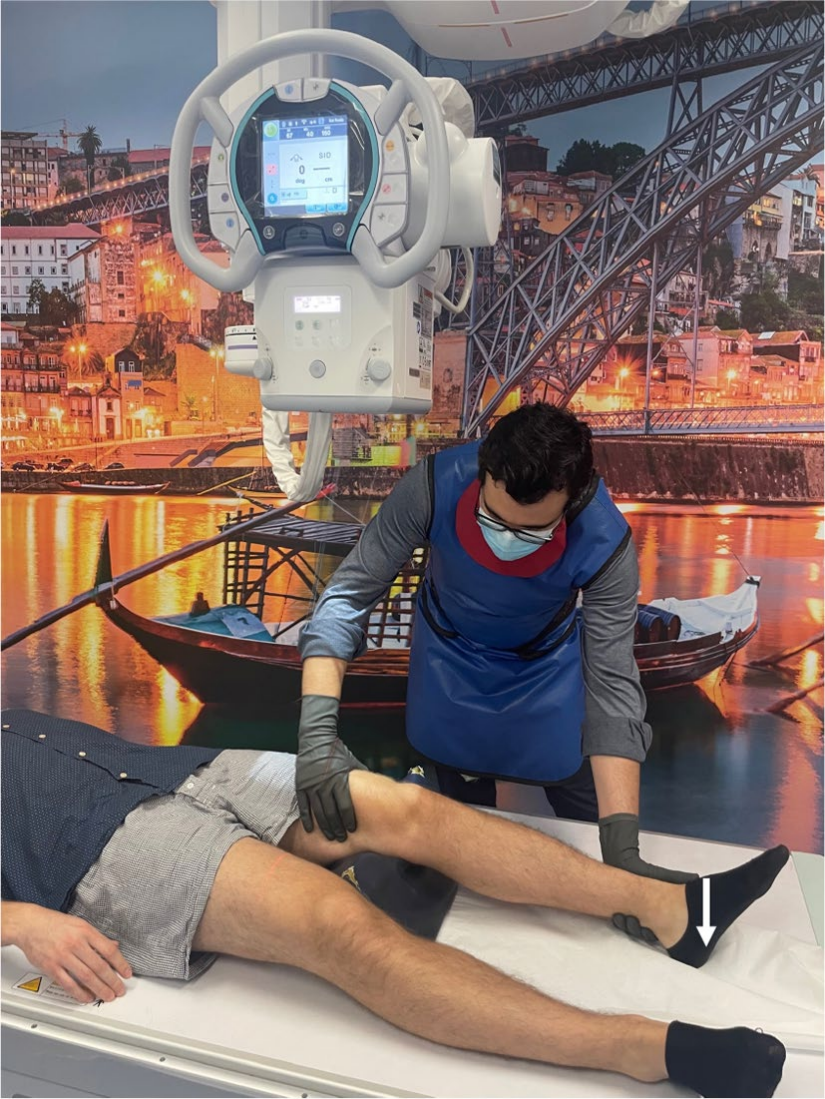
Varus stress test performed manually by a physician. The arrow indicates the movement of the hand on the tibia whilst the other hand fixes the femur

In the reviewed use case, stress radiography provides a more objective analysis of the magnitude of the knee instability, when in comparison to a physical examination [16, 22, 23, 28, 42]. Its applications cover diagnosing acute and chronic knee injuries, evaluating knee instability before and after surgery, and monitoring knee stability in non-operative treatments [22, 28, 42]. In relation to physical examination, the stress radiography of the knee may reduce the physician’s interpretation bias by providing a more reproducible and reliable examination, and giving a permanent visual record of the examination [16, 22, 23, 28, 42]. Despite stress radiography being an improvement to physical examination of knee injuries, it is still performed manually in a lot of healthcare facilities [36, 43], as shown in Fig. 3, 4, and 5. Since every manual stress radiograph exposes the physician to radiation, some physicians, like Sawant et al. [42], have created their own techniques for stress radiography to avoid exposure to radiation. However, these techniques still suffer from subjectivity and lack of reproducibility, because they all depend on the operator and the patient. In addition, these types of stress radiography leave artefacts in the X-ray images, such as the hands of the operator (Fig. 6) [43], materials used by techniques created by physicians [42], or even the table where the examination was performed (Fig. 7).

**Fig. 5:**
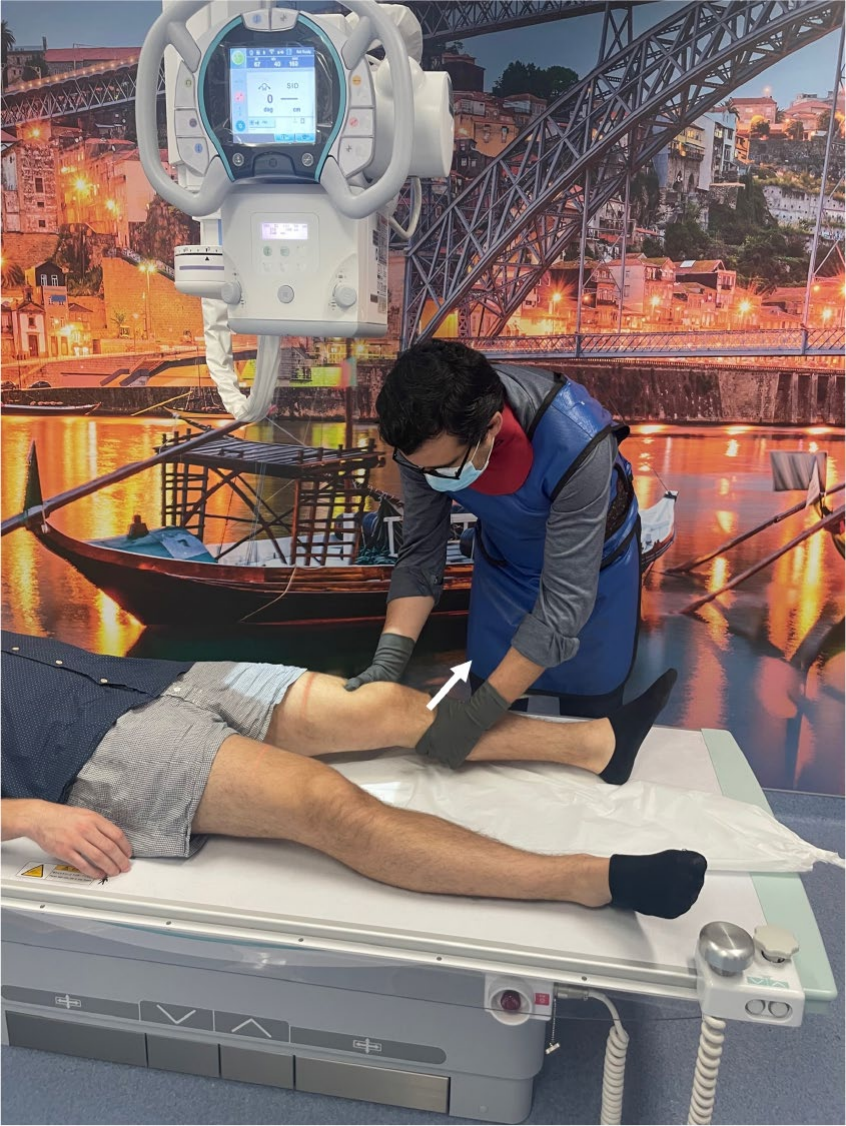
Lachman test performed manually by a physician. The arrow indicates the movement of the hand on the tibia whilst the other hand fixes the femur

**Fig. 6:**
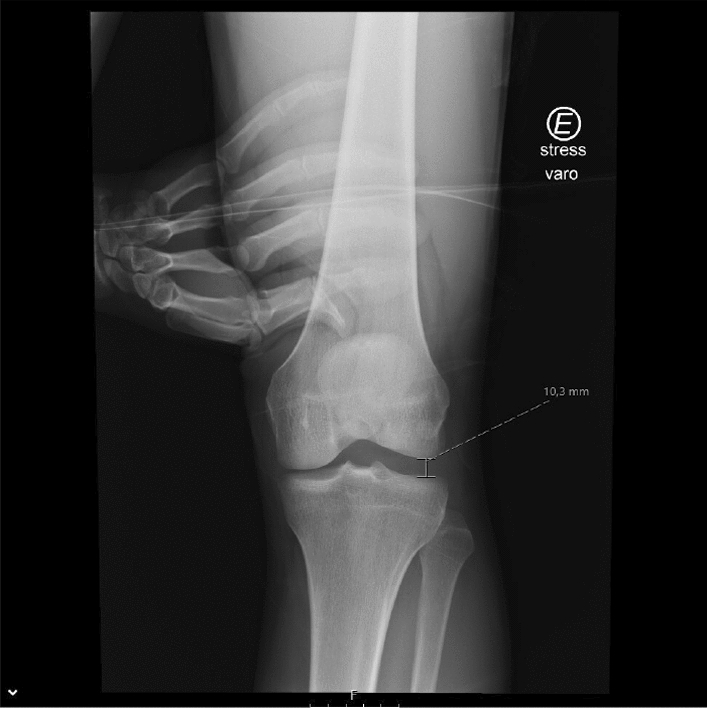
X-ray image with hands of a physician whilst performing the varus stress on the left knee (“E” is an indicator for “Left”).

**Fig. 7:**
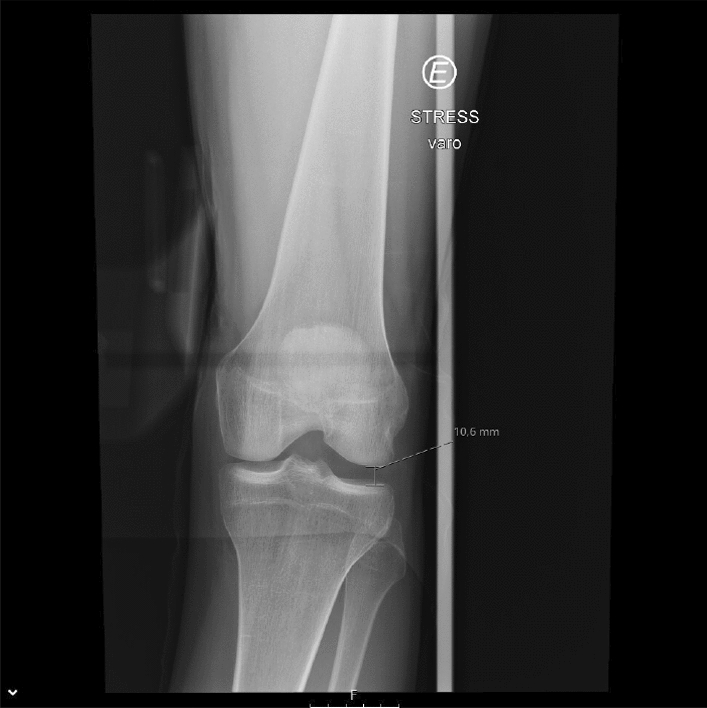
X-ray image with artefact left by the table of examination during the performance of the varus stress test on the left knee (“E” is an indicator for “Left”)

The X-ray is the primarily chosen radiographic examination by physicians, comprising over a half of the total ordered examinations, which also include CT and MRI scans. In 2018, 152.8 million X-rays out of 267.7 million imaging tests were made in hospitals in the USA [48], and 22.9 million X-rays out of 42.7 million imaging tests were performed in the UK [35]. Nevertheless, a lot of these X-rays images end up being rejected by physicians. A study at a major metropolitan emergency medical imaging department in Australia analysed a total of 90,298 X-ray images, since June 2015 until April 2017, and achieved a 19% reject rate out of 4965 knee X-rays [4]. The most common reason for rejection was inadequate patient positioning with almost an half of the number of overall rejected images [4]. Other common reasons for rejection were artefacts, which can include clothing artefacts and patient movement [4]. Such a high amount of rejected knee X-rays will have a great economic impact in the health system of each country. Rejected knee X-rays also impact the health of the patient, who had to be exposed to more radiation than it was necessary, and of the physician, in case they performed several manual stress radiographs. Even though the exposure of the patient would be quite low and harmless, the exposure of the physicians to radiation can put them at a greater risk of exceeding the annual radiation dose limits (0.05 Sv per year [31]), in case they do not practice proper radiation protection measures and adhere to safety practices [8]. Even so, being exposed so closely to radiation on a daily basis, even with personal protective equipment (PPE), could put physicians at risk for long-term adverse health effects, such as loss of white blood cells, reduction in platelets, fertility problems, and changes in kidney function [2].

This paper focuses on the review of positioning systems for knee stress radiography, including the medical background about the human knee joint. The remainder of this paper is organised as follows. “Background” section presents a brief explanation of the anatomy and stress radiography of the knee. “Methodology” section presents the methodology used for this review work. In “Knee positioning systems for stress radiography” section, all the related work with positioning systems for knee stress radiography is presented and, in “Discussion of the current knee positioning systems for stress radiography” section, it is analysed. “Limitations of the presented study” section sums up the current work with conclusions, including some future work related to functionalities that might be interesting to add to the systems. To the best of our knowledge, the works mentioned in this paper are not covered by other review documents.

## Background

This section will address some concepts that will serve as a background for this literature review. “Anatomy of the knee joint” section presents a brief analysis on the anatomy of the human knee joint, and “Stress radiography of the knee” section introduces stress radiography as a way of evaluating the knee.

### Anatomy of the knee joint

The human knee joint is a synovial joint, meaning that the articulating surfaces of the bones are enclosed in a capsule filled with synovial fluid that lubricates the movement between them [17, 25, 33]. Synovial joints are diarthrosis, since they allow freedom of movement, and can be sub-classified into various types of joints, depending on the shape of the articulating surfaces and the movement. Considering that the knee predominantly allows a rotational motion through flexion and extension of the leg, it is classified as an hinge joint [17, 19, 25, 33]. The knee joint links together the thigh bone (femur) and the shin bone (tibia). The femur has an additional articulating surface with the kneecap (patella) inside its joint capsule, while the tibia articulates with the fibula outside it [17, 19, 25, 33]. It is inside the joint capsule that the synovial membrane produces the synovial fluid, lubricating the knee and reducing friction in motion [17, 19, 24, 33]. Around the end of the bones, there is a white soft cartilage that helps the joint move smoothly, known as articular cartilage [24]. Furthermore, the knee joint has ligaments that are bands of tough elastic tissue responsible for stabilising and limiting the movement of the knee [30]. Ligaments are classified depending on whether they are enclosed by the knee joint capsule or surrounding it. There are two major extracapsular ligaments: the MCL, and the lateral collateral ligament (LCL); and two major intracapsular ligaments: the ACL, and the posterior cruciate ligament (PCL) [17, 19, 25, 30, 33].

### Stress radiography of the knee

Due to the knee joint being one of the most stressed joints of the human body and having minimal side-to-side motion, it is susceptible to injuries and degenerative joint diseases [17, 24, 33]. The diagnosis of ACL and MCL injuries and of KOA, as well as the monitoring in non-operative treatments and evaluation before and after operative treatments, usually involves the application of stress radiography [22, 28, 42]. This medical image provides a more objective analysis of the measurement of the knee laxity than a physical examination does [16, 22, 23, 28, 42].

**Fig. 8:**
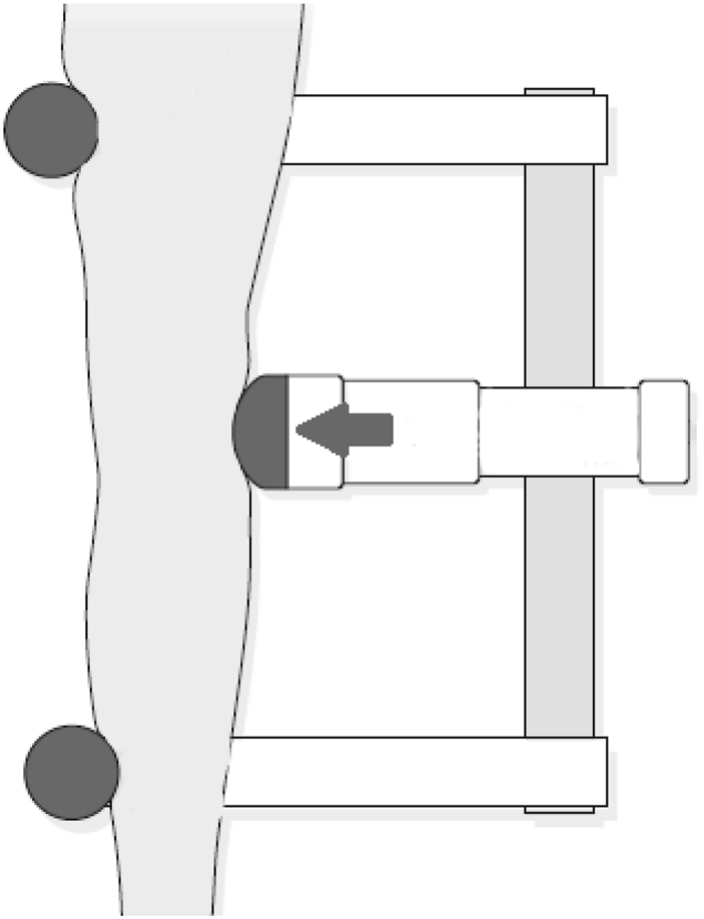
Schematic valgus stress test performed on the knee with the Telos Stress Device, Adapted from [26]

Some of the most frequent stress tests used during stress radiography to evaluate the knee laxity for KOA are the valgus and the varus stress tests [15, 23, 26]. The valgus stress test is also widely used to evaluate knee instability for injuries of the MCL [16, 27]. To obtain these types of X-ray images, the literature agrees that the patient should be positioned in supine position with a knee flexion of approximately $20\,^{\circ }$–$30^{\circ }$ by placing a radiolucent cushion under the knee [16, 23, 26, 27]. For patients with a medium height (around 1.70 m), this cushion should be about 100 mm high [16]. For application of pressure at the level of the joint-line for both tests, physicians either (subjectively) apply pressure with their hands (Figs. 3, 4) [36, 43], or use a stress device to apply a standardised force that should not exceed 150 N (Fig. 8) [23, 26, 29]. For the valgus stress, the pressure is applied on the lateral epicondyle of the distal femur, whereas for the varus stress, it is applied on the medial epicondyle of the distal femur [16]. When performing these stress tests manually, the hand that is not applying the pressure should be placed on the distal extremity of the lower limb, as seen in Figs. 3 and 4 [16]. However, when using a stress device, two supports are placed medially on the femur and the tibia, as far away from each other as possible, as shown in Fig. 8 [26].

The Lachman test is also a physical examination manoeuvre often performed in stress radiography. It is used to assess the integrity of the ACL when an injury is suspected [6, 10]. For this stress test, the patient is, once again, positioned supine with their injured knee flexed about $20^{\circ }$–$30^{\circ },$ while also slightly rotating the injured leg externally [6, 10, 13]. A force is applied to the proximal tibia either subjectively with the hand of the examiner (Fig. 5), or with a stress device with a force below 150 N [6, 10, 13]. When applying the pressure manually, the other hand stabilises the distal femur, whereas with a stress device, the two supports are placed medially on the femur and the tibia [6, 10, 13].

## Methodology

Two main steps are on the basis of this review: the search for recent related work using (knee stress radiography) as the main keywords; and the review and analysis of the collected work. During the search phase, multiple strings were created based on the main keywords to cover the largest number of papers and to prevent the exclusion of any relevant paper. The strings used were the following:

- (“knee” OR “knee joint”) AND (“stress” OR “radiography” OR “stress radiography” OR “stress X-ray”) AND (“device” OR “system” OR “positioner” OR “positioning device” OR “positioning system”);
- (“knee” OR “knee joint”) AND (“valgus stress” OR “varus stress”) AND (“device” OR “system” OR “positioner” OR “positioning device” OR “positioning system”);
- (“knee” OR “knee joint”) AND (“Lachman test” OR “Lachman stress test”) AND (“device” OR “system” OR “positioner” OR “positioning device” OR “positioning system”).

The collection of the related work was performed between October and November 2022, mainly resorting to the scientific search engines Elsevier, Springer, PubMed, Web of Science, Espacenet, and the existing websites of the positioning devices found. The search was open to any stress radiography device that assessed the human knee joint in vivo with valgus, varus or Lachman test. From this search 7 articles, 2 patents, 1 user manual and 1 website were retrieved. The assessment of the systems found and general information of the search are presented in Table 1, from the latest publication to the oldest. The analysis of the related work is limited to systems designed with the objective of performing stress radiography without the assistance of a physician or technician.

**Table 1:** Systems assessment and general information

Positioning system	Article	Publication year	Publication venue	Database
–*	Said et al. [41]	2020	Magnetic Resonance Materials in Physics, Biology and Medicine	Springer
Telos Stress Device	Koppens et al. [26]	2018	Skeletal Radiology	Springer
Laxmeter	Beukes et al. [7]	2018	Frontiers in Biomedical Devices (BIOMED)	ASME Digital Collection
–*	de Aguiar et al. [12]	2017	Arthroscopy Techniques	Elsevier
–*	Eriksson et al. [15]	2010	Knee Surgery, Sports Traumatology, Arthroscopy	Springer
–*	Beldame et al. [6]	2010	Orthopaedics and Traumatology: Surgery and Research	Elsevier
–*	Sawant et al. [42]	2003	The Knee	Elsevier
Innomed’s Self Stress Assembly Set	–**	–***	Innomed Knee Instruments—patient positioners	Innomed

*No brand name, **no articles associated, ***no year associated

## Knee positioning systems for stress radiography

Due to the lack of objectivity and reproducibility of manual stress radiography, stress radiography positioning systems have been developed. These devices help positioning the patient during the X-ray exam, whilst applying stress to a desired joint of the body.

There are several systems already in use capable of positioning the patient and applying stress to the injured joint. The Telos Stress Device is the standard device technique used for stress radiography of the knee joint. This device allows for an overall examination of the knee joint’s laxity [13], and it is capable of positioning the patient in supine position for valgus (Fig. 8) and varus stress, as well as for the Lachman test. The lower limb is secured by means of two extension arms with a counter support at the extremities of the device, which can be adjusted to the length of the leg [13]. The middle pressure part of the device has an electronic measuring equipment that is used to apply the chosen force on the joint, and to display it [13]. The device works on alkaline batteries, so the lifetime will be of approximately 150–200 h without changing them [13]. However, a cushion to flex the knee is still necessary [13]. The Telos Stress Device’s schematic patent can be found in [44]. This patent was approved in 1980, and was filed by Olaf Tulaszewski in Germany. It involves a “Leg positioning device for X-ray filming” that has a support that can fix the lower limb at two different spaced locations, a pressure part between those two locations that applies an incrementally variable pressure to the leg, and a device for the measurement of the applied force, so that it can be easily reproduced [44].

A device similar to the Telos Stress Device, created by Said et al. [41], has the objective to perform valgus and varus stress tests in both X-ray and MRI environments, consisting of a loading and a connected control unit. The loading part of the system, composed of MRI-compatible materials, uses a pneumatic actuator placed at the centre of the device. The pressured air supplying the actuator will provide the force necessary to perform the stress test. On the opposite side of the central pressure applicator, there are two adaptable counter-bearings that fix the upper and lower limb, to ensure the reproducibility of the force application. These counter-bearings and the actuator are padded with biocompatible materials, so as not to injure the skin of the patient. When it comes to the control unit, it consists of a digital-to-analogue converter and a proportional pressure valve, that is actuated electronically. These two components ensure the connection between the pneumatic actuator and the pressure outlets. Moreover, the digital-to-analogue converter is controlled by software routines that allow the pressure valve to go from 0 to 4.69 bar. As calculated by Said et al. [41], this allows the pneumatic actuator to provide a maximum force of 230.2 N.

The Laxmeter is a radiolucent stress radiography device that was initially developed to facilitate the measurement of knee joint ligament laxity at various angles of joint flexion [7]. It consists of two primary systems: an ergonomic patient support structure, and a load application system [7]. The acrylic ergonomic support structure allows the positioning of the patient at multiple degrees of knee joint flexion [37]. The respective patent was filed by the University of Cape Town, South Africa and accepted in 2015. It entails an “Anatomical support facilitating medical imaging of the hip, leg and knee” that is divided into an horizontal pelvis support panel, which is attached to an edge of a thigh supporting panel, connected to an opposite edge of a shin/foot. All these panels are made of radiolucent material. Both the pelvis support panel and the thigh panel have anchorage attachments to restrain these parts of the body of the patient. Furthermore, there are locking sections in the shin/foot support panel to adjust the structure into different positions parallel to the pelvis support panel [37]. The Cast-Nylon electromechanical load applicator applies an incremental translational force necessary to perform various stress tests, including valgus, varus, and Lachman, and is complete with pressure sensing pads [7]. This load varies from 0 to 250 N in 25 N increments, being held for 30 s in each position to allow for X-ray image capturing, and assessing the anterior, posterior, medial, and lateral parts of the proximal lower leg [7]. The lower limb is supported by the ergonomic structure and by velcro straps. Since the Laxmeter is meant for laxity measurement via stress radiography, it has the integration of a stress radiography bone translation tracking system that gives real-time diagnostic information [7]. This tracking system involves a radiopaque scale embedded onto the ergonomic support that is visible on stress radiographs [7].

A varus/valgus stress device was developed by Eriksson et al. [15] to create a constant stress force on the knee joint without the need of an operator in the examination room. This system was capable of adjusting a force up to 10 kg to apply a valgus or varus stress test, whether in full extension of the leg or at $30^{\circ }$ flexion [15]. Despite the literature not providing much information about the specifications of the device, by analysing [15] it is possible to assume the pressure is applied to the tibia and there is only one support that fixes the upper limb with the force of the patient’s foot.

Innomed’s Self Stress Assembly Set is a simple positioning system for stress radiography that is already on the market. It consists of a triangle positioner and a contoured cube, where the triangle positioner is used as an external cushion to flex the patient’s knees; and the contoured cube is positioned between the patient’s feet. The pressure for the stress tests is applied by the compression of the contoured cube by the patient’s feet [21].

Other systems in the literature provide simple and economical solutions to avoid the exposure of the physician to radiation. It is the case of the valgus stress technique for stress radiography applied by Sawant et al. [42], where the knees are bound together using a firm 6-in. crepe bandage, a cassette is placed longitudinally under both knees, and the physician stands at the feet of the patient applying a force to both feet. Another example is a technique used to apply the valgus stress test during stress radiography by de Aguiar et al. [12]. This technique created by physicians uses a similar approach to the Sawant et al. [42] technique, bounding the knees together using a velcro belt placed around the patellar level, and keeping the medial part of the upper limbs together. A wedged material made of ethylene vinyl acetate is placed with its larger side below the malleolar level of the ankle, much like it is done with the Innomed’s Self Stress Assembly Set, and the two opposing forces create a valgus stress to the knees. In case the physician decides to perform a valgus stress with the knee at $30^{\circ }$ flexion, then a soft pad, also made of ethylene vinyl acetate, can be placed under the knee. Finally, there is a technique used for the Lachman test during stress radiography by Beldame et al. [6], where weights are wrapped around the patient’s foot whilst they are sitting on half of their lower limb. In this case, the patients have to keep themselves in the Lachman position and contract their own quadriceps to produce the anterior drawer of the tibia [6].

To the best of authors knowledge, and after using the methodology mentioned in “Methodology” section, there are no other knee positioning systems in the literature that perform stress radiography using the valgus or varus stress, or Lachman test in vivo. From all of the positioning systems mentioned before, only the Telos Stress Device and the Innomed’s Self Stress Assembly Set are currently on the market, showing that there is a clear gap in this area of orthopaedics.

## Discussion of the current knee positioning systems for stress radiography

Although the Telos Stress Device is used worldwide for stress radiography of most human joints (e.g. knee, ankle, wrist, elbow), being able to perform stress tests, such as the valgus and varus stress, and Lachman test, it still has some limitations. This popular device is not radiolucent, leaving artefacts of the support and pressure parts in the X-ray images [6, 26]. These artefacts could make the X-ray image more difficult to read to the physician, since it is quite distracting and does not give the plain anatomy of the joint. Furthermore, the Telos Stress Device needs an external cushion to provide the flexion of the knee joint in order to perform the stress tests.

Despite being an objective and reproducible positioning system, that functions not only in a radiographic environment, but also in a MRI environment, the system proposed by Said et al. [41] does not have radiolucent materials, generating artefacts in the obtained X-ray images. Besides, it requires an external cushion in case the physician desires to provide flexion to the knee during the stress radiographs.

The Laxmeter already provides the flexion of the knee with its ergonomic support structure, not requiring an external cushion. However, it is limited to the knee joint, not being able to assess other joints, and the force is given by increments, which makes it take longer if the operator wants to assess only one part of the knee. Moreover, it has a radiopaque scale for laxity measurement, that is visible in stress radiographs. Even though this radiopaque scale would allow the physician to assess the injury in real time, most physicians do not find this necessary for diagnosis, preferring a clean X-ray image. In addition, this device is a big, heavy-weight, and unfoldable structure that could have a negative impact in health facilities, delaying the stress radiography set-up.

Furthermore, the varus/valgus stress device developed by Eriksson et al. [15] is able to assess the varus and valgus knee stress tests by using an objective and reproducible method, but it is not able to address the Lachman test nor other joints. In addition, it seems to need an external cushion for the flexion of the knee, and the support of the upper limb seems to be a radiopaque material [15].

Other stress radiography devices fail at providing the objectivity and reproducibility that are needed. The Self Stress Assembly Set requires the patients to perform the valgus stress themselves, giving subjectivity to the system, since an injured patient will not apply the pressure needed after they start to feel pain. In addition, this system can not support the extremities of the lower limb, which might lead to motion artefacts and incorrect positioning on the X-ray images. Some simpler and more economic solutions in literature avoid the physician to be in direct contact with radiation, but still are based on subjective techniques that provide no fixed positioning of the patient.

A comparison between the features of all the mentioned knee positioning systems and their limitations is represented in Table 2. Despite the Telos Stress Device being the most well-established on the market, it fails to be radiolucent and ergonomic, since it is made of radiopaque materials and needs an external cushion to flex the knee. In addition, physicians have mentioned that this device is too confusing to set-up, ending up not utilising it in a clinical setting. The Laxmeter does not address more than one joint and it is a big heavy structure that ends up not being of simple use, nor radiolucent. The device created by Said et al. [41] and Eriksson et al. [15] are not radiolucent nor ergonomic, failing to assess more than one joint. The Self Stress Assembly Set does not have objectivity and reproducibility, and it is not able to assess more than one joint. Finally, the simple technique invented by Sawant et al. [42] has no benefits, whereas the ones used by de Aguiar et al. [12] and Beldame et al. [6] are radiolucent.

**Table 2:** Comparison of the features of the knee positioning systems and their limitations

Features	Positioning system							
	(Said et al.)	Telos Stress Device	Laxmeter	(de Aguiar et al.)	(Eriksson et al.)	(Beldame et al.)	(Sawant et al.)	Self Stress Assembly Set
Objectivity/reproducibility	$\checkmark$	$\checkmark$	$\checkmark$	$\times$	$\checkmark$	$\times$	$\times$	$\times$
Radiolucent	$\times$	$\times$	$\times$	$\checkmark$	$\times$	$\checkmark$	$\times$	$\checkmark$
Ergonomic	$\times$	$\times$	$\checkmark$	$\times$	$\times$	$\times$	$\times$	$\checkmark$
More than one joint	$\times$	$\checkmark$	$\times$	$\times$	$\times$	$\times$	$\times$	$\times$

The methods of all the knee positioning systems mentioned in “Knee positioning systems for stress radiography” section are compared in Table 3. When setting up the device to perform the chosen stress test, only the Laxmeter has an automatic set-up, whereas the other devices require it to be done manually. The force is applied to the joint through automatic methods that do not require the presence of an operator in the examination room except for the Self Stress Assembly Set, and the techniques used by Sawant et al. [42], de Aguiar et al. [12], and Beldame et al. [6], that require a manual force to be applied to/by the patient. Regarding whether the positioning device is equipped with sensors or not, the Self Stress Assembly Set, and the techniques used by Sawant et al. [42], de Aguiar et al. [12], and Beldame et al. [6] do not have any sensors, since these systems are manual. The existence of sensors in the technique created by Eriksson et al. [15] is uncertain. When it comes to the type of stress tests addressed by the positioning systems, only the Telos Stress Device and the Laxmeter are capable of performing the valgus, varus, and Lachman tests. The techniques created by Said et al. [41] and Eriksson et al. [15] perform both the valgus and the varus stress tests, whereas the remaining analysed systems can only perform one type of stress test.

**Table 3:** Comparison of the techniques of the knee positioning systems

Techniques	Positioning system							
	(Said et al.)	Telos Stress Device	Laxmeter	(de Aguiar et al.)	(Eriksson et al.)	(Beldame et al.)	(Sawant et al.)	Self Stress Assembly Set
Set-up of stress test	Manual	Manual	Automatic	Manual	Manual	Manual	Manual	Manual
Type of force	Automatic	Automatic	Automatic	Manual	Automatic	Manual	Manual	Manual
Use of sensors	$\checkmark$	$\checkmark$	$\checkmark$	$\times$	?	$\times$	$\times$	$\times$
Type of test	Valgus/varus	All	All	Valgus	Valgus/varus	Lachman	Valgus	Valgus

After the analysis of Tables 2 and 3, it is clear that there is a need for a knee positioning system that has all of the features that physicians expect from one of these systems: objectivity/reproducibility, being radiolucent and ergonomic, and assessment of more than one joint, never forgetting this type of medical device needs to be safe, reliable, and of simple use. The device also needs sensors for the automatic set-up of the stress test and the automated force. Moreover, it is important that the system is capable of addressing more than one type of stress test, in order to be more efficient.

## Limitations of the presented study

The presented review has its limitations. Even though there are more systems in the literature used for performing valgus/varus stress or the Lachman test, the majority of those systems are used for laxity measurements of the knee when a stress test is applied to the joint. With that in mind, the authors decided to focus on systems that were designed with the sole objective of positioning the knee for stress radiography without the help of a physician or technician, in order to conclude whether there was a need for the healthcare system for this type of device. Furthermore, there is little to no information about the systems presented in “Knee positioning systems for stress radiography” section, since the authors might not wish to disclose the specifications of the devices, in case they decide to put them out on the market.

## Conclusions and future work

The knee is the human joint most prone to injuries, and physicians find it difficult to carry out an objective assessment of its pathologies. Knee stress radiography provides a more objective analysis of the laxity of an injured knee joint. However, the devices available to perform different diagnosis of knee injuries, evaluation of knee instability before and after surgery, and monitoring of knee stability during non-operative treatments have limitations. In order to improve these medical devices, new knee positioning devices have been developed.

In spite of that, a radiolucent solution for positioning of the patient’s knee during stress radiographs without the assistance of a physician during the X-ray examination is in need for healthcare professionals. With such a tool, the number of knee X-rays rejected by physicians would decrease, since there would be less inadequate positioning and movement from the patient. With the decrease in X-rays there would be not only a decrease in costs for the healthcare system, but also a faster and more reliable examination of knee pathologies, allowing the workflow of healthcare facilities to run more smoothly. In addition, healthcare professionals would decrease their exposure to radiation and the X-ray image would not have artefacts of their hands, making it easier to be analysed. This solution should have objectivity/reproducibility, be radiolucent, ergonomic, of simple use, safe and reliable, and be able to assess more than one joint. Furthermore, the positioning system needs to have sensors to ensure that it is an objective/reproducible system that lacks dependency on the operator, and addresses more than one type of stress test.

## Data Availability

The authors affirm that human research participants provided informed consent for publication of the images in Figs. 2a, b, 3, 4, 5, 6, and 7.
